# Application of Multiprotocol Medical Imaging Communications and an Extended DICOM WADO Service in a Teleradiology Architecture

**DOI:** 10.1155/2012/271758

**Published:** 2012-02-06

**Authors:** George V. Koutelakis, George K. Anastassopoulos, Dimitrios K. Lymberopoulos

**Affiliations:** ^1^Wire Communication Laboratory, Department of Electrical and Computer Engineering, University of Patras, 26504 Patras, Greece; ^2^Medical Informatics Laboratory, Medical School, Democritus University of Thrace, 69100 Komotini, Greece

## Abstract

Multiprotocol medical imaging communication through the Internet is more flexible than the tight DICOM transfers. This paper introduces a modular multiprotocol teleradiology architecture that integrates DICOM and common Internet services (based on web, FTP, and E-mail) into a unique operational domain. The extended WADO service (a web extension of DICOM) and the other proposed services allow access to all levels of the DICOM information hierarchy as opposed to solely Object level. A lightweight client site is considered adequate, because the server site of the architecture provides clients with service interfaces through the web as well as invulnerable space for temporary storage, called as User Domains, so that users fulfill their applications' tasks. The proposed teleradiology architecture is pilot implemented using mainly Java-based technologies and is evaluated by engineers in collaboration with doctors. The new architecture ensures flexibility in access, user mobility, and enhanced data security.

## 1. Introduction

Digital Imaging and Communication in Medicine (DICOM) network protocols are widely used for reviewing images and performing primary diagnosis within radiology and other imaging departments, by means of Picture Archiving and Communication System (PACS). DICOM, which is the communication standard for medical imaging, is also applied in teleradiology cases, where the transfer of patient radiological images from one location to another for the purposes of interpretation and/or consultation is performed. In teleradiology, professionals belonging to different departments of the hospital (internal professionals) or professionals at home (external professionals) communicate with the radiology department.

In these cases [1], DICOM network protocols are less frequently used to share DICOM Objects for diagnostic purposes, especially between hospital departments and external professionals. Image communication is not performed strictly for diagnosis only but also for educational, scientific, and other co-operational activities of external professionals. In these activities, they have lower expectations of image quality and reliability [1]. They desire more flexible applications, which can be integrated with their other desktop applications and less centralized system setup and configuration. This paper focuses on teleradiology performed by external professionals through the Internet.

DICOM Supplement 54 (DICOM-E-mail) [1] suggests a connection between the Internet and hospital medical communication. In [2], an example of DICOM protocols' vendor-specific addition for the implementation of certain teleradiology solutions is demonstrated. Moreover, a variety of common Internet protocols, that is, HTTP, FTP, SMTP, IMAP4, POP3, as well as vendor-dependent protocols are evidently acceptable in certain teleradiology solutions [3–5].

Surveys in different countries, for example, Switzerland [6], have shown that users demand different protocols (Switzerland Survey: DICOM 52.4%, HTTP 26.2%, e-mail 21.4%, and FTP 14.3%—from source [6]) to support teleradiology services, because this is more convenient for them. Moreover, a strong focus on standards such as DICOM and HL7, as well as on the results of the IHE Initiative, indicates that different protocols, and the possibility of converting between them, are demanded if flexible software architecture is to be created that can be adapted to user requirements [7].

The Internet evolution has also shown how to integrate medical imaging services over Internet platforms, such as portals [7–9]. Until now, several integrated platforms, referred to as “web PACS” or “web teleradiology,” have been launched by many developers, either as research results or as products [10–12]. These platforms allow remote access to medical imaging services using light client applications and web browsers instead of conventional heavy PACS terminals. Of course, PACS terminals are still used inside radiology departments. However, the obvious benefits of user mobility are compensated by the demand for potential better medical image quality or the comprehensiveness of image processing tools. The early Internet solutions were not well acceptable from radiologists due to their limitations. However, the broadband networks and the high software technology have eliminated these limitations. The future in teleradiology software is the Internet-based software.

Besides Supplement 54, Web Access to DICOM Persistent Objects (WADO), which is another DICOM Internet extension, allows the access and the presentation of “DICOM Persistent” Objects (reports, waveforms, images, etc.) using DICOM Unique Identifiers (UIDs). WADO employs web protocols (HTTP/HTTPs) instead of the tight DICOM message exchange protocols [13]. In [14], the authors propose a WADO service extension called the “Web Access to DICOM Archives (WADA)” service. The WADA service extends WADO access capability to the whole DICOM hierarchy (Patient, Study, Series, and Object), includes an additional internal query mechanism, and supports the web submission of medical reports.

This paper presents a set of medical imaging services provided to remote users for the purposes of interpretation or consultation. Such an independent teleradiology system can also work as a PACS' augmented system [15, 16]. This work considers teleradiology functionality to be the aggregation of the WADA service as well as other DICOM and conventional Internet services (i.e., web, FTP, and E-mail). These services are combined to provide an integrated operational environment for user interaction with the medical archives. This functionality allows access to all levels of the DICOM Archives' hierarchy instead of limiting it to Objects, through multiprotocol communications and web interfaces.

“Full” user mobility and enhanced security demands have led to the adoption of a three-tier teleradiology architecture, including client, middle (server), and data tier. In compatible teleradiology and PACS architectures, the temporary storage and viewing of any retrieved image examination areperformed by resources of the client site. In the proposed architecture, the middle tier performs storing and processing operations on behalf of clients and provides the client tier with all special viewing/reading facilities. The middle tier provides users with the web interfaces of all supported services, any dedicated purpose application, and invulnerable personal storing domains (thereafter called User Domains), which are visible from anywhere through the web. Moreover, it ensures enhanced security due to the transfer of files between User Domains and PACS Archives without the client's interpolation.

Hence, off-the-shelf personal computers, without installing any specific enterprise software application and any type of lightweight client structures, are adequate in the client tier.

The design of the new architecture is compatible with other existing architectures in the field of healthcare information systems and allows the addition of more modules related to new operations or services.

In the following sections, the design of the overall three-tier teleradiology architecture is presented, including the WADA service, the User Domains and their services, as well as collaboration services. A pilot implementation of the proposed multiprotocol teleradiology architecture and services is also demonstrated enhanced with advanced security mechanisms. Finally, a discussion-evaluation regarding the implemented teleradiology architecture is presented.

## 2. The Multiprotocol Teleradiology Architecture

**Figure 1:**
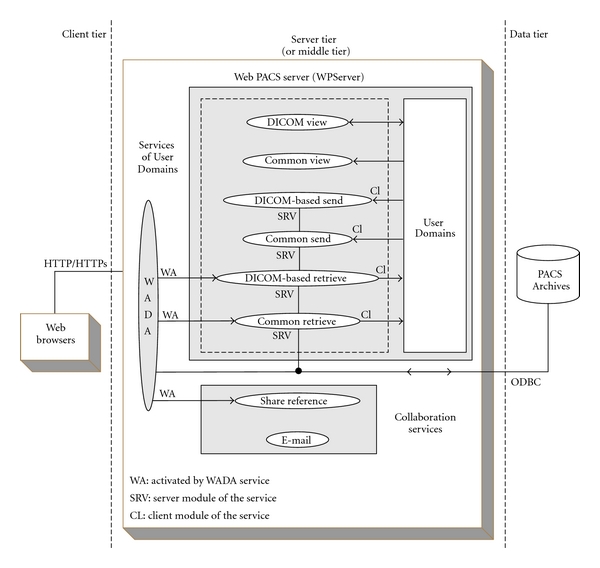
The three-tier multiprotocol architecture of the new teleradiology platform.

Figure 1 depicts the proposed three-tier multiprotocol architecture that composes a new teleradiology platform. The data tier comprises the PACS Archives, including the databases (data, metadata, and enterprise management rules) which store all attributes related to the DICOM Information levels, and the whole file system of the Object files. The server tier comprises the Web PACS Server (WPServer). The WPServer interacts with the data tier through the Open Database Connectivity (ODBC) protocol and provides the client tier with the graphical user interfaces (GUIs) of services through standard web communication, employing HTTP/HTTPs protocol. The client tier includes all different types of web browsers supporting any request to access middle tier services. The three-tier architecture preserves data security and integrity since the archived data are accessed by the clients only through the server tier. The clients are not in direct contact with the data tier. The users have no knowledge of the underlying procedures cooperating in order for the user to obtain data.

The WPServer is the most important and complicated part of this architecture. The WPServer delivers all the services provided to web clients, processes service requests coming from the client tier, and responds properly, providing users with all the necessary information through dedicated interfaces. 

The services integrated into the WPServer accept clients' requests, form the suitable queries, and access the PACS Archives to select or update stored information, employing either a hierarchical or a relational query model depending on the request. The hierarchical model requires the UIDs of all the previous levels of the DICOM hierarchy and the UID of the queried level, whereas the relational model requires only the UID of the queried level. Each teleradiology platform service accomplishes a set of actions, cooperating to ensure the same capability. Each service can integrate modules, which operate either individually or in pairs through a client-server communication mode. In the case of a client-server pair, the server module is able to access the PACS Archives through an ODBC connection. The sections below describe the services provided by the WPServer. 

### 2.1. WADA Service

The DICOM standard organizes the medical image examinations under the entities Patient, Study, Series, and Composite Object, all referred to as Information Entities (IEs). These IEs are hierarchically related, by means of Information Models (IMs), for the purpose of implementing DICOM services.

The WADA service [14] adopts the Patient-Root IM handling access to the whole of the DICOM IEs. WADA also adopts two operational modes, “direct” and “dynamic.” 

The “direct” mode shares medical images by structuring web references at all DICOM IEs. The UIDs of the requests are provided by external procedures. The response on request of Patient, Study, and Series level is a table including all requested IE instances withall related attributes for each one of them. The response on an Object level request is the requested Object. The web references of “direct” mode can be applied inside documents and e-mail messages.

The “dynamic” mode enhances the “direct” mode with an internal query mechanism calling consecutive processes that allows only one UID per process to be requested. This mechanism is a standard-compatible procedure, based on the query concept of DICOM C-FIND service [17, 18], to obtain the required UID field of the HTTP structured request for the IE instances. Each kind of UID is used for the retrieval of the corresponding DICOM entity's instance. Next, the server demonstrates the instance to the client, which has the ability to proceed through the next query. The first query is always sent to the Patient level to support the baseline hierarchical query mechanism [17]. The next query is sent to the Study level and so on until the Object level. The response to each level request is similar to the “direct” mode. In web-based medical systems, the “dynamic” mode should be implemented and operate as an independent image enabler in medical IT infrastructures, such as PACS, RIS, EMR, and telemedicine-teleradiology.

The WADA service also provides authorized users with the ability to submit medical reports associated with the requested Study/Series for remote diagnosis workflows, that is, urgent medical incidents. A medical report composition-submission form provides remote clients with web-based diagnosis interaction. WADA introduces a new procedure that includes the UID of the Series or the Study in the query field of the HTTP request.

The WADA service transforms DICOM formats of the Objects into presentation-ready formats, for example, jpeg, html. The Objects of response can be in proper Multipurpose Internet Mail Extension (MIME) type [13] including the “native” DICOM and other common data formats.

Table 1 depicts the general structure of WADA requests. In both WADA modes, the request types “WADA” and “WADA-R” provide access to information and report submission, respectively. Table 1 also illustrates the request formats of “direct” and “dynamic” WADA, including examples. It is noted (regarding Table 1) that Koutelakis et al. [19] conclude that the uniqueness of a Patient inside a group of hospitals that pull Patient ID from the same pool is ensured by the compound key of the Patient ID, Patient Name, and Patient Birth Date attributes. This group of attributes will hereafter be referred to as “Patient UID.”

A combination of the “direct” and the “dynamic” modes is also available for users. In this “hybrid” mode, the user first accesses the Patient, Study, or Series level through the “direct” mode and then accesses the lower level instances through the “dynamic” mode.

### 2.2. Services of User Domains

**Figure 2:**
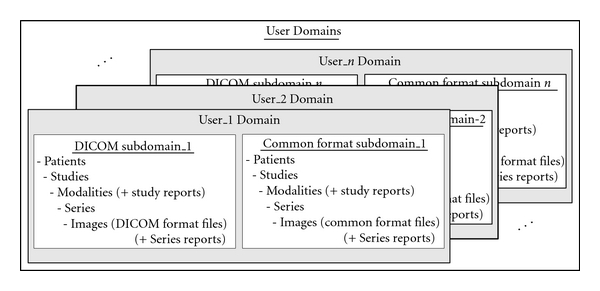
The structure of the User Domains.

User Domains (Figure 2) comprise a file system used for temporary storage or preview of Object files (images, reports, and waveforms) under process by a specific user. The centralization of User Domains inside the WPServer is considered to ensure convenience of web mobility. Additionally, it provides extra security in DICOM communication (through web interfaces) since the Object files are never transferred to the client's site but between the PACS Archives and the User Domains.

Each User Domain allows only authorized access and browsing through web interfaces. It is divided into two different subdomains and has a tree-like directory structure. The first subdomain stores files of Objects containing images and waveforms of native DICOM format. The second subdomain stores files of Objects containing images and waveforms of common formats supported by WADA (e.g., jpeg, gif). The reports are stored in both subdomains.

In the denominating of directories belonging to both subdomains, “matter-of-fact” names are used, instead of “full” UIDs suggested by DICOM. This is for user convenience. The allocation of directory names adopts the Patient-Root IM hierarchy, enhanced with the Modality level. Hence, for the hierarchical organization of Object storage, Patient ID, Study ID, Modality types (MR, CR, CT, US, NM, etc.), and Series ID are employed as directory names. These IDs are often not unique outside the range of a hospital, however, they are short numbers, easily remembered, and very useful in the process of ordering the restricted number of Patients and Studies stored in a User Domain each time. Modality level is added to distinguish between the Series of one Study produced by modalities located in different sections of a radiology department.

User Domains provide the advantage of user mobility. The main shortcoming of User Domains is the lower speed of image processing, because images are located in the WPServer. This drawback is eliminated in broadband networks. Another shortcoming can be the need for maintenance related to User Domains, when the system does not offer enough storage space. However, this shortcoming is easy resolved by ensuring a large storage space, proper administrating rules, and system administrator surveillance.

User Domains include a set of services related to the transfers of medical imaging examinations to and from the User Domains. These services are described below.

#### 2.2.1. DICOM-Based Retrieve Service

It fetches DICOM Objects from the PACS Archives and transfers them to a User Domain, based on C-GET/C-STORE DICOM messaging [18]. It integrates a client module related to User Domains and a server module related to PACS Archives. The client and server modules communicate each other employing the C-GET/C-STORE DICOM messaging. The DICOM-based Retrieve service is activated by a request process from the WADA service, which finds a specific IE instance with the related Objects in DICOM format. Afterwards, the DICOM Retrieve service determines the corresponding Objects of this instance that have to be fetched. This service is enabled on demand after the performance of the WADA service. Depending on the IE instances handled, there are four operations of this service.

- *DICOM Patient Retrieve*. This fetches all DICOM Objects related to one Patient instance.
- *DICOM Study Retrieve*. This fetches all DICOM Objects related to one Study instance.
- *DICOM Series Retrieve*. This fetches all DICOM Objects related to one Series instance.
- *DICOM Object Retrieve*. This fetches one specific DICOM Object.

This service provides users with web interfaces to employ DICOM communication for retrieval within the whole DICOM hierarchy.

#### 2.2.2. Common Retrieve Service

It fetches common format Objects from the PACS Archives and transfers them to a User Domain, based on the GET method of the FTP service. It integrates a client module related to User Domains and a server module related to PACS Archives. The client and server modules communicate each other employing the FTP messaging. The Common Retrieve service includes four partial operations which are in full correspondence with the DICOM-based Retrieve service. This service provides users with the capability to retrieve a large number of common format medical images without overloading an E-mail server.

#### 2.2.3. DICOM-Based Send Service

It sends all DICOM Objects belonging to one IE instance directory from a User Domain (sub-domain of DICOM Objects) to the PACS Archives. It integrates a client module related to User Domains and a server module related to PACS Archives. The client and server modules communicate each other employing the C-STORE DICOM messaging system [18]. The DICOM-based Send service consists of five different operations corresponding to the five levels of a User Domain.

- *Patient DICOM-based Send*. This enables the DICOM Send service for each Object belonging to one Patient directory.
- *Study DICOM-based Send*. This enables the DICOM Send service for each Object belonging to one Study directory.
- *Modality DICOM-based Send*. This enables the DICOM Send service for each Object belonging to one kind of Modality directory (for the above Study).
- *Series DICOM-based Send*. This enables the DICOM Send service for each Object belonging to one Series directory.
- *Object DICOM-based Send.* This enables the DICOM Send service for the selected Object only.

This service provides users with web interfaces to employ DICOM communication for sending within the whole DICOM hierarchy. It is required in order to send the whole set of images belonging to one IE instance to another user of the system or to send processed evidence images to the PACS Archives.

#### 2.2.4. Common Send Service

It sends all common format Objects, which belong to one IE instance directory, from a User Domain (sub-domain of common format Objects) to the PACS Archives or to a remote web client. It integrates a client module related to User Domains and a server module related to PACS Archives or to the remote web client. The Common Send service employs the PUT method of FTP service to transfer Objects to the PACS Archives. Inside the PACS Archives, an additional procedure takes care of the proper archiving of the incoming Objects. Common Send also provides users with the capability of attaching Objects to e-mail messages and it employs SMTP protocol for the transfer of messages to remote users. The Common Send service includes five partial operations which are in full correspondence with the DICOM-based Send service. This service provides users with the ability to send a large number of common format evidence medical images to the PACS Archives without overloading an E-mail Server or to use E-mail protocols for sharing complete sets of images for the purpose of collaborative diagnosis.

#### 2.2.5. DICOM View Service

It displays and processes DICOM single-frame and multiframe images stored inside the DICOM sub-domain and acquired from various modalities (e.g., X-ray, CT, MRI, CR, angio, US, etc.). It allows the use of 2D image processing functions (e.g., zoom in/out, magnifier, window level, smooth/sharp, annotations, pseudo color, etc.) to enhance the client diagnostic capability. The activation of this service requires additional functionality from the user web browser, downloaded from the WPServer after the request for this service. This service offers facilitations related to the viewing or processing of “native” DICOM medical images.

#### 2.2.6. Common View Service

It displays common format images that are stored inside the Common Format sub-domain. The activation of this service does not require any additional functionality, but only a web browser. It is used for expeditious or routine diagnostic performance. This service provides the capability of flexible viewing of common format medical images for a routine diagnosis and for educational, scientific, or any other purpose that does not require best quality medical imaging but greater flexibility.

### 2.3. Collaboration Services

The WPServer also includes two services that enhance the collaboration between the users of the teleradiology platform.

#### 2.3.1. Share References Service

It shares references of archived IE instances (Patient, Study, Series, and Object) with collaborative users. References formatted in “direct” WADA mode are loaded inside the body of an e-mail message that is shared with users. It is activated on demand after a WADA service request process which finds the specific IE instance. This service offers the capability of sharing “direct” WADA references through E-mail protocols during the browsing of Patients' examinations, eliminating any complexity related to the user side procedure and providing motivation for user collaboration in viewing and reporting a medical examination.

#### 2.3.2. E-Mail Service

It provides all common storing, communicating, creating, and viewing functions of an E-mail service, which pass through an external E-mail Server. It is the asynchronous communication mode for the enhancement of user collaboration. This service also offers the capability of image transfer from clients between users. However, the outside images, stored in clients' site, are not kept in the User Domains or the PACS Archives. The system administrator can manage these images manually. Users can review these images and reply to sender through e-mail messages. This practice ensures that all the stored images in the overall teleradiology platform come from the PACS Archives of strictly authenticated imaging centers and that these images are archived in complete sets belonging to the corresponding IE instance.

## 3. Implementation of the Architecture

**Figure 3:**
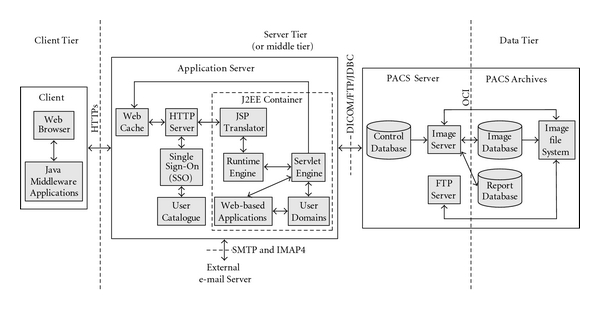
The operational components in the implementation of the teleradiology platform architecture.

Figure 3 illustrates the operational components in the pilot implementation of the proposed teleradiology platform architecture. The client tier is implemented by desktop, laptop and tablet PCs, which are capable of running a Web Browser and Java Middleware Applications as the Java 2 Runtime Environment (J2RE). The server tier (WPServer) is implemented by two discrete servers: the Application Server and the PACS Server. In the server tier, components are also applied that handle authorization and encryption issues regarding the teleradiology platform. The PACS Archives of the data tier are implemented by two separate databases—the Image Database and the Report Database—as well as the Image File System that keeps the files of Images. 

### 3.1. Implementation of the Application Server

**Figure 4:**
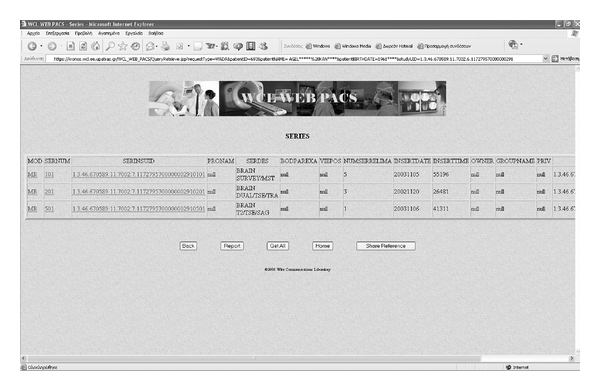
User interface of the WADA service—Series table of a Study.

**Figure 5:**
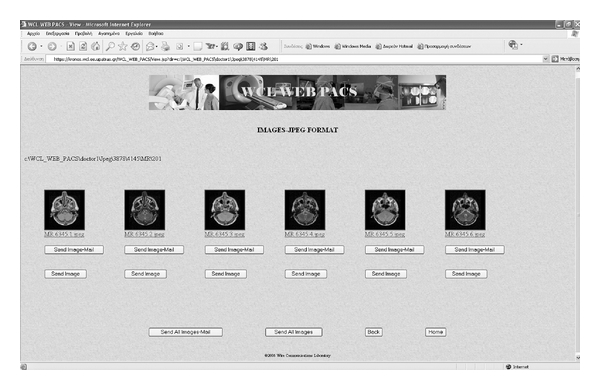
User interface of the Common View service—Thumbnails of images belonging to a Series.

**Figure 6:**
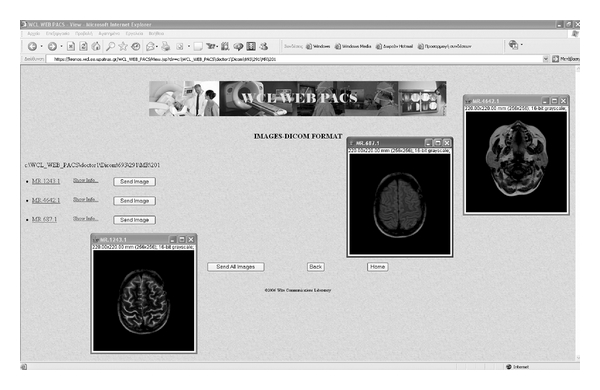
User interface of the DICOM View service—Java applets embedded in JSP.

The Application Server is implemented to handle the whole of the teleradiology platform services provided to the client tier except from the DICOM server modules and FTP server modules. The modules of services, which are integrated into the Application Server, are implemented with Java libraries, JSPs, Java applets embedded in JSPs, and JavaBeans and are integrated into the Web-based Applications, deployed within this server. The Web-based Applications support all interactions between the Application Server and the client tier through the distinguished presentation (static) and application logic (dynamic) parts of the web pages (Figures 4 and 5). Java applets, embedded in JSPs, are used only for the implementation of the DICOM View service (Figure 6).

Java applets are supported by J2EE Container and run at the client tier by means of a web browser using Java Virtual Machine (JVM) embedded within the J2RE application installed on the client tier PCs. JSPs are selected due to their advantage to create distinguished static and dynamic (JavaBean) parts within a page.

Java DICOM libraries of the Pixelmed DICOM toolkit which is offered by the official FTP site of the Medical NEMA Organization contribute the classes of most DICOM services and WADO. The WADO libraries are modified to support the new WADA service that is proposed in this paper. The Pixelmed libraries are also modified to support Oracle database. XML-based tools of Pixelmed are used in the implementation of medical report structuring and in other parts of the service application. The use of Java API for XML Processing (JAXP) ensures XML support. Communication between the Java applications of the Application Server and the databases of the DICOM Archives is implemented through the registration of the Oracle Java Database Connection (JDBC) drivers. JavaMail library and JavaBeans Activation Framework (JAF) support the development of E-mail applications. The bzip2 compression algorithm is also integrated into transfer applications development. The integration of bzip2 reduces both network transfer time and stored-memory usage.

The JSP processing environment is built to be compatible with the suggestions posed by the Apache consortium. It consists of the JSP Translator, the Runtime Engine, and the Servlet Engine integrated within the Java-2 Enterprise Edition (J2EE) Container. The User Domains are deployed inside the J2EE Container in order for Object files to be integrated into the Container along with Web-based Applications.

The employed JSP Translator and Runtime Engine are built to provide full support for JSP Directives, all core/standard JSP tags and embedding Structured Query Language (SQL) within Java. The Runtime Engine is used to distinguish the dynamic part from the static part. HTML code supports the static part of the page and Java code configures the dynamic part, which is data originating from user entries or server resources and which extends HTML capabilities. The Servlet Engine processes the dynamic JSP part by forwarding requests (either locally to the Web-based Applications and User Domains or remotely over Internet Protocol—IP to the PACS Server) and waiting for replied data. The JSP Translator, the Runtime Engine, and the Servlet Engine are built with the technology of the Oracle Application Server.

Web Cache and HTTP Server are also integrated into the Application Server to support Web-based Applications. They handle the allocation of all user requests and the response operations. The Web Cache stores frequently access web pages in memory, eliminating the need for repeated process requests on the HTTP Server. If the requested pages are not cached, the Web Cache forwards the requests to the HTTP Server, which is based on Apache web technology. Next, if the requests come from an authenticated user, the HTTP Server forwards them for response structuring. The Web Cache and the HTTP Server are built with Oracle Application Server technology.

### 3.2. Implementation of the PACS Server and the Archives

The PACS Server is implemented to handle transfer and storage of images or Studies (with or without report) between the data tier (Image File System, Image Database, and Report Database of PACS Archives) and the Application Server (User Domains). It is the DICOM and FTP service provider of the Application Server, because PACS Server integrates DICOM server modules and FTP server modules. The PACS Server implements and deploys the FTP Server and DICOM Server.

FTP protocols are implemented by the FTP Server is embedded in the Solaris operating system of the Intel Pentium server. FTP Server components of the operating system have been activated. DICOM protocols are implemented by the CTN software (Central Test Node) Image Server, following suitable modifications required to support Oracle databases and medical report transfers. CTN is free open-source software for DICOM services provision. It has been developed in C programming language by the Washington University in St. Louis [20]. The operation of DICOM services' modules is supported by a relational Control Database, implemented with Oracle. The Control Database is a lookup table with all information required during DICOM communication. This information concerns IP address, port, Application Entity (AE), and supplementary optional data units related to the DICOM communication entities. Each User Domain is considered an independent DICOM communication entity, in which a unique AE is allocated in order to perform all DICOM communication services.

The Image and Report Databases of the PACS Archives are designed as relational databases and are implemented with the Oracle Database Management System. They interact with the Image Server through the Oracle Call Interface (OCI), which includes a set of subroutines called by applications implemented in C programming language. The whole implementation of these databases is based on the concept of CTN [20]. The Image Database consists of Patient, Study, Series, Image, and Image-Instance tables relating to image information. The first four tables represent all attributes of the corresponding DICOM IE. The Image-Instance table represents the attributes of the Image file that is the Image IE in the physical medium. There is one-to-one correspondence between attributes and the table columns. The files of medical images in either DICOM or common format are archived within an integrated Image File System, which is located beside the Image Database. This file system has the structure of the file system of User Domains (reports excluded) and interacts with both the Image Server and the FTP Server. The Report Database contains tables for storing the content of report tags as diagnostic text (“Interpretation” and “Results”), identification of the physician/user who created the report, record, and approval date/time, the UIDs of the Study, Series, and Objects that were reviewed for the report creation, and so forth. The UIDs constitute the association link between the report and the related (reviewed) Study, Series, and Objects from the Image Database. Notice that both databases are used for simultaneous temporal and permanent Objects storage due to the limited scale of this implementation.

### 3.3. Security Issues

Web-based DICOM applications inherently introduce security problems. DICOM protocol structurally supports only client-server applications where the client is in direct contact with the PACS Server. The Internet technology adopts the three-tier architecture, where PACS Server is invisible to the client. Client and PACS Server communicate through an intermediate server named the Application Server, which ensures an adequate security level through two communication stages. The first (insecure) stage is between the clients and the Application Server. The second (secure) stage is between the Application Server and the PACS Server.

The proposed teleradiology architecture performs two-stage communication in one stage, since the User Domains are located in the Application Server. This architecture allows the PACS Server to receive only files created inside the overall system of the servers—no external file can be transferred to it. Hence, medical information is not transferred to the client side, so both the security of the system and the mobility capabilities of the user are increased. Additional secure communication between the clients and the Application Server is ensured through the implementation of communication encryption and user identification issues. 

*Communication encryption* between web clients and the Application Server is implemented over the secure HTTPs protocol. The installed HTTPs is configured to transmit data in encrypted form using a 128-bit key size for the stream encryption algorithm. This is considered an adequate degree of encryption for medical data exchanges, according to the Health Insurance Portability and Accountability Act (HIPAA) organization.

*User identification* is implemented using the Single Sign-on (SSO) procedure specified in the OASIS standard [21, 22]. Access to the accounts of the different User Domains and to the provided applications is permitted through the SSO. A single authenticated session across multiple-enable services and applications is also permitted by the SSO. The utilization of the authenticated session allows the client to perform secure operations with any enabled service within the system. The user authentication state is tracked by cookies, which are used by the SSO to pass information to each web resource. The implemented SSO component, on a first layer, ensures the user identification process through a log in screen that requires a unique id and a unique password for any user to enter. On a second layer, the SSO accomplishes the whole logical process that is responsible for translating a user request into applications logging. The SSO and User Catalogue utilize a cross-reference procedure. The users already registered in the Catalogue are authorized to continue navigation inside the application platform. The employed User Catalogue implements the Lightweight Directory Access Protocol (LDAP), version 3.

Both HTTPs and SSO are built with Oracle Application Server technology.

## 4. Discussion-Evaluation

The proposed teleradiology services have been designed as a result of close collaboration between medical doctors and engineers. Doctors' demands related to imaging exams and the corresponding medical reports were analyzed. This required analysis contributed to the design of additional teleradiology services in addition to those included in DICOM standard, in order to satisfy real-world framework demands. This analysis also featured the innovations integrated into the design of the illustrated teleradiology platform.

The developed Application and PACS Servers were installed for pilot use in the Intranet of the Patras University Campus. A group of technical and medical experts evaluated the proposed teleradiology services using ADSL network connection (wired or wireless). Two hospitals (University Hospital of Patras and the Olympion private hospital in the city of Patras) participated in the evaluation procedure. The authorized clients established Virtual Private Network (VPN) client software for access to the teleradiology platform VPN. The VPN client software was available for downloading after user identification in the support area of the teleradiology platform. The pilot operation scenarios included the following.

- Reviewing radiological Studies stored in hospital storage from private practice offices.
- Reviewing the results of a Study, for which they were the referral doctors through the internal hospital Ethernet and the ADSL connection between hospitals and the University Campus.
- Doing homework as employees of a hospital.

During the six-month period of the pilot operation phase, 152 Patients, 654 Studies, and 312 medical reports were archived in the PACS Archives. The overall size of the archived data inside the PACS Server exceeded 10 GB. Users downloaded nearly 100 GB of data into the User Domains. They were advised to delete redundant data from their User Domain because the hard disks of the Application Server used in the pilot operation phase had a restricted total capacity of only 146 GB.

The architecture presented describes a center-based implementation of image sources. The PACS Archives are shown to be the only image source of the teleradiology platform. External files of images can be transported between clients as attachments using the E-mail service of the teleradiology platform. These files are kept out of the PACS Archives and the User Domains.

Independent medical imaging centers that use standardized archives can be considered to be additional PACS Archives, allowing the adding of files to the central system. In the implementation presented, these centers should incorporate open databases for JDBC connections. If these centers incorporate closed databases (not allowing JDBC), they could be provided with DICOM Store or FTP services implemented in the teleradiology platform. The latter case requires additional implementation customization. In this paper, JDBC solution is chosen for avoiding data duplication in the Application Server, thus eliminating the implementation scale and complexity.

The entirety of the images comes from strictly authenticated imaging centers (established as PACS Archives). This “limitation” favours the constitution of a complete and approved Patient folder including complete radiological Studies and not different sets of images. This “limitation” also deters users from sending incomplete sets of images, overloading the archiving system, and confusing any other user.

The submission of any external report is allowed through the submission form of a proper “WADA-R” request, which includes text areas for filling. The file of a report is always created by the system itself. The open source implementation regarding the medical report based on CTN and Pixelmed efficiently covered the entirety of the demands. However, the DICOM Structured Reporting (SR) solution can be easily integrated into the presented open architecture for system enhancement. One of the future plans is to upgrade medical report implementation employing DICOM SR.

It should also be noted that, nowadays, the IHE Cross-Enterprise Document Sharing-Imaging (XDS-I) integration profile [23] seems to be the most promising attempt for an integrated medical image sharing standard, as the ARTEMIS research project concludes [24, 25]. However, the complexity of the XDS-I makes the integration of XDS-I into existing medical IT systems difficult. The use of XDS-I in such systems requires the redesigning of their architecture in each case to be based on XDS-I.

The web-based software components of the teleradiology platform construct an open architecture. They can also be easily installed or integrated into any other independent medical IT platform (already constructed or established) to offer comprehensive tools for interaction with the archived medical imaging examinations through the web. The XDS-I may be the future for medical imaging communications. However, because of its sophisticated architecture, the XDS-I concept should be well considered before the design and implementation phase of any new medical IT system. Otherwise, such a new system cannot be integrated into XDS-I.

## 5. Conclusions

Throughout the described teleradiology system, the main target has been the integration of services that ensure flexibility in the whole DICOM archive access, “full” mobility for users, and enhanced security for data. The flexibility in the archive access is accomplished by the new WADA service and other medical imaging communication services based on a multiprotocol infrastructure. The whole of these services offer access to the whole DICOM archive rather than solely to Objects. The use of the WADA requests inside other services (e.g., Share Reference service) amplifies the above flexibility. “Full” mobility is accomplished providing users with—apart from web-based service interfaces—personal User Domains for temporary storage of images and reports enabling them to fulfill application tasks from anywhere in the world without the necessity of using their own PCs. Users do not need to transfer medical data for processing to the local PC but keep it inside the Application Server. This part of the architecture also enhances the security of the data, which is transferred within the overall teleradiology platform as users cannot upload local data from their PCs to the PACS Server directly. The whole procedure of editing or previewing images and reports is performed by users through their User Domains. Additional security is employed for communication encryption and user identification, ensuring the privacy of medical images and reports. The aggregation of provided services within this flexible, mobile, and secure environment is designed to satisfy most radiologist's and clinician's demands, loading client PCs with no specific operational, computing, or storing requirement.

This architecture is implemented using mainly Java-based technology as well as other reliable standard-compliant products, which guarantee accurate performance. The modular structure allows the cooperation with existing architectures in the field of healthcare and the easy adaptation of additional components according to user demands.

The DICOM standard, which is the dominant protocol for medical imaging communications, provides a new concept for communication service design. It accepts E-mail service and adopts web-enabled services through the standardization of WADO service. The proposed teleradiology platform architecture is a new system based on this concept.

## Figures and Tables

**Table 1:** WADA requests.

Type	Request for:	URL of requests
General type of WADA	**WADA**: Access to Information **WADA-R**: Report Submission	<scheme>://<authority><path>?<requestType=WADA (or WADA-R)> &<UIDS=<uids≫ &<optionals=<values≫

“Direct” WADA Mode (Samples)	Patient table	*https://kronos.wcl.ee.upatras.gr/WCL_WEB_PACS/QueryRetrieve.jsp?requestType=WADA *
	Study table of a Patient	https://kronos.wcl.ee.upatras.gr/WCL_WEB_PACS/QueryRetrieve.jsp?requestType=WADA &patientID=693&patientNAME=AGEL****%20KAN****&patientBIRTHDATE=1961****
	Series table of a Study	https://kronos.wcl.ee.upatras.gr/WCL_WEB_PACS/QueryRetrieve.jsp?requestType=WADA &patientID=693&patientNAME=AGEL****%20KAN****&patientBIRTHDATE=1961**** &studyUID=1.3.46.670589.11.7002.6.117279570000000291
	Submission of a new report related to a Series	https://kronos.wcl.ee.upatras.gr/WCL_WEB_PACS/QueryRetrieve.jsp?requestType=WADA-R &patientID=693&patientNAME=AGEL****%20KAN****&patientBIRTHDATE=1961**** &studyUID=1.3.46.670589.11.7002.6.117279570000000291 &seriesUID=1.3.46.670589.11.7002.7.1172795700000002910101

“Dynamic” WADA Mode (Samples)	Patient table	*https://kronos.wcl.ee.upatras.gr/WCL_WEB_PACS/Patients.jsp?requestType=WADA *
	Study table of a Patient	https://kronos.wcl.ee.upatras.gr/WCL_WEB_PACS/Studies.jsp?requestType=WADA &patientID=693&patientNAME=AGEL****%20KAN****&patientBIRTHDATE=1961****
	Series table of a Study	*https://kronos.wcl.ee.upatras.gr/WCL_WEB_PACS/Series.jsp?requestType=WADA &studyUID=1.3.46.670589.11.7002.6.117279570000000291 *
	Submission of a new report related to a Series	*https://kronos.wcl.ee.upatras.gr/WCL_WEB_PACS/Objects.jsp?requestType=WADA-R &seriesUID=1.3.46.670589.11.7002.7.1172795700000002910101*
